# Acute Hepatitis and Pancytopenia in Healthy Infant with Adenovirus

**DOI:** 10.1155/2016/8648190

**Published:** 2016-06-01

**Authors:** Amr Matoq, Asma Salahuddin

**Affiliations:** ^1^Department of Pediatrics, University of Florida, College of Medicine, Jacksonville, FL 32207, USA; ^2^Department of Pediatrics, Division of Hospital Pediatrics, University of Florida, College of Medicine, Jacksonville, FL 32207, USA

## Abstract

Adenoviruses are a common cause of respiratory infection, pharyngitis, and conjunctivitis in infants and young children. They are known to cause hepatitis and liver failure in immunocompromised patients; they are a rare cause of hepatitis in immunocompetent patients and have been known to cause fulminant hepatic failure. We present a 23-month-old immunocompetent infant who presented with acute noncholestatic hepatitis, hypoalbuminemia, generalized anasarca, and pancytopenia secondary to adenovirus infection.

## 1. Introduction

Adenovirus, a DNA virus, is a common cause of febrile illness in children. It presents mainly with respiratory symptoms. And it can also present with gastrointestinal symptoms, conjunctivitis, or cystitis. It is usually self-limited, but, in immunocompromised hosts, it has been associated with more severe illness. Adenoviruses are transmitted via contact and droplet route. There is no specific treatment; supportive management should be provided according to the symptoms. There is no available vaccine for the general public. Adenoviruses have been reported as a cause of acute hepatitis in immunocompromised patients; however, only few cases were reported in immunocompetent hosts. We describe a case of an immunocompetent infant, with acute hepatitis and pancytopenia secondary to adenovirus.

## 2. Case Presentation

A previously healthy 23-month-old female presented with a two-day history of rhinorrhea, cough, decreased energy, sleepiness, and tactile fever. On the day prior to admission, she had two episodes of nonbloody, nonbilious emesis and several episodes of nonbloody diarrhea; she also developed generalized swelling of the face, arms, and legs.

She had recently completed a ten-day course of Bactrim for a urinary tract infection with the last dose being five days prior to admission. She initially presented to an outside medical center where labs were consistent with hepatitis and she was transferred to our facility for further management. Her mother denied any recent travel, sick contacts, or decreased urine output. She was up to date with her vaccinations including hepatitis A vaccine, and she had unremarkable past medical and perinatal history. Family history was negative for liver disease.

Physical exam was remarkable for a fussy but consolable infant, with mild bilateral conjunctival injection and dry lips. Scattered crackles were audible on auscultation of lungs bilaterally, with no tachypnea or retractions. Cardiac exam revealed mild tachycardia, with no murmur or gallop. Generalized edema was noted including periorbital, bilateral pedal, and labial edema. There was no evidence of jaundice, and neurologically she had intact mentation with no concern for encephalopathy. Labs revealed elevated transaminase AST of 889 IU/L and ALT of 740 IU/L and albumin of 2.2 g/dL with prolonged INR of 1.5, PTT of 55 seconds, normal bilirubin, and normal electrolytes. Initial CBC showed mild leucopenia of 3.5 K/microL and anemia Hgb of 10 g/dL; this progressed to pancytopenia on days 2 and 3 with platelets down to 77 K/microL with low reticulocyte count. Ferritin and LDH were high, as expected for inflammatory process. By day 5, patient transaminases have markedly improved, and pancytopenia had resolved (Table 1).

Urinalysis was unremarkable, and blood culture and urine culture were negative. Stool analysis, stool culture,* Helicobacter pylori* antigen, and* Clostridium difficile* toxin were all negative. Acetaminophen level was normal. Viral hepatitis serology and CMV were negative. EBV IgG was positive, and IgM was negative. Celiac screen was negative. CXR showed bilateral mild pleural effusion. Echocardiography showed 2-3 mm small pericardial effusion. Ultrasound of the abdomen showed enlarged echogenic liver and pericholecystic fluid and gall bladder sludge. Nasopharyngeal secretion viral testing was positive for adenovirus. The test used was Hologic (Prodesse) ProAdeno+ Assay, which is the qualitative detection of human adenovirus (HAdV) serotypes 1–51 DNA.

### 2.1. Hospital Course

**Figure 1:**
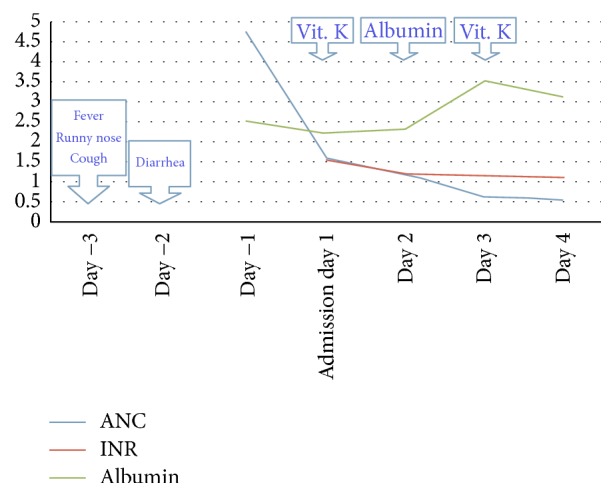
Hospital course diagram. ANC: absolute neutrophil count; INR: international normalized ratio. The *x*-axis represents units for albumin (g/dL) and ANC (K/microL).

Supportive care was provided. The patient remained afebrile. Due to the worsening swelling and edema, albumin 25% was administered. Due to prolonged INR, vitamin K replacement was given for three days. The patient's edema subsequently improved, cell lines and liver function had improved, and the patient was discharged five days later (Figure 1).

### 2.2. Final Diagnosis

The patient was diagnosed with acute hepatitis, with decrease in synthetic function of the liver and pancytopenia attributed to adenovirus infection. This is consistent with the patient's history of upper respiratory symptoms and fever two days prior to the generalized swelling and a negative workup for other common causes of hepatitis and pancytopenia.

## 3. Discussion

We report a previously healthy 23-month-old patient with acute noncholestatic hepatitis, with hypoalbuminemia and anasarca and pancytopenia associated with adenovirus infection. Adenovirus hepatitis has been described in immunocompromised patients including transplant patients [1] and those with malignancy [2]. Only few cases of adenovirus hepatitis in immunocompetent patients have been reported [3–6].

Adenoviruses are common respiratory viruses responsible for 5 to 10 percent of all febrile illnesses in infants and young children [7]. Multiple serotypes have been identified; they are transmitted via respiratory and fecal-oral routes and show seasonal variation with wintertime peak [5, 6]. Fever is present in almost all patients [5, 6], with upper respiratory infection, pharyngitis, keratoconjunctivitis, enteritis [8], and hemorrhagic cystitis being common manifestations.

Generally, adenoviruses infection is self-limited; however, disseminated infection can occur even in immunocompetent hosts [3]. A retrospective review by Rocholl et al. [6] reported one immunocompetent case of fulminant hepatic failure with encephalopathy among 143 adenovirus positive cases. Özbay Hoşnut et al. [4] reported an 18-month-old infant with fatal fulminant hepatitis, found to be secondary to adenovirus. Liver involvement and elevation of LFTs have been studied in a retrospective study by Peled et al. [5] and were found to be more frequent in younger children. In the same study, 39 out of 78 adenovirus positive cases had hepatomegaly. In all reported cases of adenovirus hepatitis, diagnosis was made after exclusion of all other common causes of hepatitis such as hepatitis A, hepatitis B, and hepatitis C and CMV and EBV.

Our patient had also completed a course of Trimethoprim-Sulfamethoxazole (TMP-SMX). TMP-SMX induced hepatitis has been reported and studied in adults especially in HIV patients on PCP prophylaxis [9–12]. There are also few reports on pediatric patients [13–17]. Fever, rash, and eosinophilia were described in most of the cases. There are several liver-specific causality assessment scales. Standard liver-specific Council for International Organizations of Medical Sciences/Roussel Uclaf Causality Assessment Method (CIOMS/RUCAM) scale is widely used [18]. Using this scoring system, our case scores 5, which indicates a possible adverse drug reaction. Our patient had no evidence of eosinophilia or rash. Although TMP-SMX reaction cannot be excluded, it is less likely in our case.

Subclinical elevation of LFTs is known to occur frequently with EBV [19] and occasionally with adenoviruses [5]; however, acute hepatitis with generalized anasarca is seldom seen. Onset of the swelling in this case was two days after the fever and URI symptoms; thus, the onset of hepatitis was likely concomitant with the respiratory symptoms.

Our case report highlights to clinicians the importance of including adenoviruses infection in the differential diagnosis of acute hepatitis, even in healthy immunocompetent patients, when other common causes are excluded.

## Figures and Tables

**Table 1:** Hospital course, lab results. ^*∗*^ND: note done.

	Day −1	Day 1	Day 2	Day 3	Day 4	Day 5
AST (10–60 IU/L)	889	692	827	367	165	ND
ALT (14–54 IU/L)	740	613	673	452	376	ND
Albumin (3.4–5.1 g/dL)	2.5	2.2	2.3	3.5	3.1	ND
PT (11.8–15 sec)	ND	18.1	15.9	ND	14.4	ND
INR	ND	1.5	1.2	ND	1.1	ND
PTT (22.9–37.8 sec)	ND	55.5	47.5	ND	41.4	ND
WBC (6–17.5 K/microL)	5.5	3.49	4.54	3.87	5.12	4.85
Absolute neutrophil count (1.5–8 K/microL)	4.7	1.58	1.19	0.63	0.54	690
Hemoglobin (10.5–14.2 g/dL)	10.5	10.3	10.1	9.1	9.6	9.8
Eosinophil % (0–7%)	0.8	1	2	3	2	2
Platelet (150–450 K/microL)	216	176	130	93	77	106
